# Azelnidipine, but not amlodipine, reduces urinary albumin excretion and carotid atherosclerosis in subjects with type 2 diabetes: blood pressure control with olmesartan and azelnidipine in Type 2 diabetes (BOAT2 study)

**DOI:** 10.1186/s13098-015-0073-9

**Published:** 2015-09-17

**Authors:** Kazuhito Tawaramoto, Hideaki Kaneto, Mitsuru Hashiramoto, Fumiko Kawasaki, Fuminori Tatsumi, Masashi Shimoda, Shinji Kamei, Michihiro Matsuki, Tomoatsu Mune, Kohei Kaku

**Affiliations:** Department of Diabetes, Endocrinology and Metabolism, Kawasaki Medical School, 577 Matsushima, Kurashiki, 701-0192 Japan; Takarazuka Daiichi Hospital, Takarazuka, Japan; Department of Internal Medicine 1, Kawasaki Medical School Hospital, Kurashiki, Japan

## Abstract

To evaluate the efficacy of azelnidipine and amlodipine on diabetic nephropathy and atherosclerosis, we designed a prospective and randomized controlled clinical study in type 2 diabetic patients with stable glycemic control with fixed dose of anti-diabetic medication. Although there was no difference in blood pressure between both groups, urinary albumin excretion and maximum carotid intima-media thickness were reduced in azelnidipine group, but not in amlodipine group. In addition, inflammatory cytokine levels were decreased only in azelnidipine group which possibly explains such beneficial effects of azelnidipine on urinary albumin excretion and carotid atherosclerosis.

Dear Editor,

It is well known that both type 2 diabetes and hypertension lead to the onset of cerebrovascular and cardiovascular events via the augmentation of inflammation and the progression of atherosclerosis. Therefore, physicians have to choose the most appropriate medication for the prevention of such complications. Diabetic nephropathy is also one of the life-threatening major complications and its progression is accelerated in the presence of hypertension [1, 2]. Angiotensin receptor II blockers (ARBs) are recommended as a first medication to treat hypertension in patients with type 2 diabetes, and calcium channel blockers (CCBs) are often selected as a second anti-hypertensive medication. Among many CCBs, azelnidipine is known to possess unique effect such as higher anti-inflammatory effect. Therefore, we hypothesized that azelnidipine is more effective to delay the progression of atherosclerosis and/or diabetic nephropathy, and designed a prospective, two-arm, randomized controlled clinical study with azelnidipine and amlodipine. This study was conducted with outpatients in division of Diabetes, Endocrinology and Metabolism in Kawasaki Medical School. The study protocol was approved by Institutional Review Board of Kawasaki Medical School, and the study was conducted in accordance with the Declaration of Helsinki.

We enrolled a total of 38 subjects with type 2 diabetes whose blood pressure was not sufficiently controlled by treatment with 20 mg/day of olmesartan for over 2 months. Insufficient control was defined as follows: systolic blood pressure >130 mmHg and/or diastolic blood pressure >80 mmHg according to the guideline by Japanese Society of Hypertension. Patients who signed an informed consent prior to this trial were randomly divided in azelinidipine (16 mg/day) and amlodipine group (5 mg/day). We evaluated the effects of azelnidipine and amlodipine after 32 weeks. Patients who met inclusion criteria were as follows: patients with type 2 diabetes and without well-controlled blood pressure by treatment with olmesartan; 40 < age < 75 years old; hemoglobin A1c (NGSP) is below 9.4 %. The following patients were excluded: with suspiciously secondary hypertension or non-diabetic kidney disease; with congenital dyslipidemia or extremely high LDL-cholesterol (>200 mg/dl); with advanced hepatic disease (AST > 75 IU/ml) or advanced renal failure (Crn > 2.0 mg/dl for male, Crn > 1.5 mg/dl for female); in pregnancy; with current history of any malignant neoplasm; with using steroids, any hormonal medications, any diuretics, immune-suppressing medications and/or potassium drug; with past history of stroke or myocardial infarction occurring within past 6 months from the start of the trial. There was no dropout during the follow-up period.

Carotid IMT was measured using Aplio ultrasonography (Toshiba), and images of carotid artery were scanned by computer and analyzed using IntimaScope software (MEDIA CROSS). Plasma MCP-1 (R&D systems), TNF-α (R&D systems), HMW adiponectin (R&D systems), leptin (R&D systems), soluble vascular cell adhesion molecule-1 (VCAM-1) (R&D systems) and soluble intercellular adhesion molecule-1 (ICAM-1) (RayBiotech) were measured using enzyme-linked immunosorbent assay kits. All values were expressed as mean ± s.e.m. We used the Mann–Whitney U test for unpaired data and the Wilcoxon signed-rank test for paired data.

There was no difference between the two groups in any clinical background including age, BMI, duration of diabetes, blood pressure and biochemical laboratory data except for heart rate (Table 1). Heart rate was higher in azelnidipine group. In addition, there was no difference between the two groups about treatment of any anti-diabetic or anti-dyslipidemic medication (Table 1). In both groups, systolic and diastolic blood pressure were significantly reduced during the trial (*p* < 0.05), but the efficacy of lowering blood pressure was similar (Fig. 1a). Heart rate was significantly decreased only in azelnidipine group (*p* < 0.05), although we cannot exclude the possibility that the difference of the effects on heart rate was influenced by the difference at baseline and there was not statistically significant difference in heart rate between the two groups at follow-up point.

**Table 1:** Baseline clinical data in azelnidipine and amlodipine groups

	Azelnidipine (n = 19)		Amlodipine (n = 19)		*P*
	Before	After	Before	After	
Gender (male/female)	9/10	ND	8/11	ND	
Age (years)	59 ± 2	ND	63 ± 2	ND	n.s.
BMI (kg/m^2^)	25.8 ± 1.0	ND	27.1 ± 1.0	ND	n.s.
Durations of DM (years)	9.2 ± 2.3	ND	11.3 ± 2.2	ND	n.s.
Systolic BP (mmHg)	149 ± 2.4	128 ± 2.9	148 ± 2.9	129 ± 3.3	n.s.
Diastolic BP (mmHg)	80 ± 2.4	68 ± 2.3	84 ± 2.3	72 ± 2.1	n.s.
Heart rate (beats/min)	75 ± 2.8	71 ± 2.5	68 ± 2.3	69 ± 3.5	<0.05
HbA1c (NGSP) (%)	6.9 ± 0.2	6.9 ± 0.2	7.2 ± 0.2	7.3 ± 0.3	n.s.
Fasting plasma glucose (mg/dl)	126 ± 6.4	128 ± 12.3	120 ± 4.6	130 ± 7.5	n.s.
Total cholesterol (mg/dl)	200 ± 6.3	196 ± 7.4	207 ± 6.1	196 ± 5.5	n.s.
HDL cholesterol (mg/dl)	57 ± 3.0	55 ± 3.3	55 ± 3.1	55 ± 2.2	n.s.
LDL cholesterol (mg/dl)	117 ± 6.8	113 ± 6.5	122 ± 4.9	113 ± 5.1	n.s.
Triglyceride (mg/dl)	129 ± 13.4	136 ± 18.0	144 ± 11.4	142 ± 13.4	n.s.
Creatinine (mg/dl)	0.69 ± 0.06	0.76 ± 0.04	0.70 ± 0.04	0.69 ± 0.04	n.s.
BUN (mg/dL)	17.3 ± 1.1	17.1 ± 0.9	17.2 ± 0.8	17.9 ± 0.7	n.s.
eGFR (ml/min/1.73 m^2^)	75.2 ± 3.6	73.2 ± 3.6	76.9 ± 3.2	78.2 ± 3.3	n.s.
Urinary albumin excretion (mg/g.Cr)	274 ± 202	121 ± 69	136 ± 70	202 ± 114	n.s.
Uric acid (mg/dl)	5.7 ± 0.4	5.7 ± 0.3	5.5 ± 0.3	5.5 ± 0.4	n.s.
Na (mEq/l)	141 ± 0.4	141 ± 0.5	141 ± 0.4	140 ± 0.5	n.s.
K (mEq/l)	4.4 ± 0.1	4.5 ± 0.1	4.2 ± 0.1	4.2 ± 0.1	n.s.
Cl (mEq/l)	105 ± 0.4	104 ± 0.5	105 ± 0.5	104 ± 0.4	n.s.
average IMT (mm)	0.817 ± 0.054	0.759 ± 0.034	0.873 ± 0.063	0.814 ± 0.080	n.s.
Max IMT (mm)	1.102 ± 0.101	0.908 ± 0.004	1.203 ± 0.113	1.124 ± 0.143	n.s.
Other medications					
Sulfonylurea (%)	7 (36.8)		7 (36.8)		
Metformin (%)	6 (31.6)		7 (36.8)		
Pioglitazone (%)	9 (47.4)		8 (42.1)		
α-glucosidase inhibitors (%)	4 (21.1)		2 (10.5)		
Insulin (%)	1 (5.3)		2 (10.5)		
Fibrate (%)	2 (10.5)		3 (15.8)		
Statins (%)	8 (42.1)		7 (36.8)		

*ns* not significant (azelnidipine vs amlodipine at baseline)

*p* < 0.05 azelnidipine vs amlodipine at baseline

**Fig. 1:**
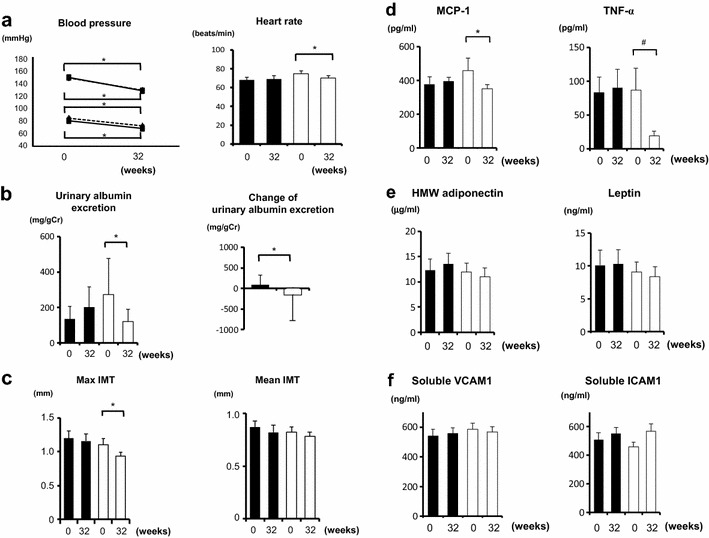
**a** Blood pressure and pulse rate in amlodipine and azelnidipine groups. Urinary albumin excretion (**b**) and carotid intima-media thickness (IMT) (**c**) in amlodipine and azelnidipine groups. Inflammatory factors (**d**), adipocytokines (**e**) and soluble adhesion factors (**f**), in amlodipine and azelnidipine groups. *Closed square* and *bar*, amlodipine (n = 19); *open triangle* and *bar*, azelnidipine (n = 19). *p < 0.05, ^#^ *p* = 0.055

At baseline, there was no difference in glucose and lipid metabolism between the two groups (Table 1). HbA1c, fasting plasma glucose and fasting plasma insulin levels were not altered during the treatment with azelnidipine or amlodipine (Table 1). Plasma triglyceride, LDL-cholesterol and HDL-cholesterol levels were not also altered (Table 1). At baseline, there was no difference in urinary albumin excretion between the two groups. Urinary albumin excretion was significantly reduced after azelnidipine treatment (*p* < 0.05), but not after amlodipine treatment (Fig. 1b, left). Urinary albumin excretion in azelnidipine group was significantly lowered compared to amlodipine group (*p* < 0.05) (Fig. 1b, right), although we cannot exclude the possibility that the difference of the effects on urinary albumin excretion was influenced by the difference of mean value in urinary albumin excretion at baseline. Max IMT was significantly decreased only in azelnidipine group at 32 weeks (*p* < 0.05), although average IMT was not altered in both groups (Fig. 1c).

Inflammation is regarded as a key factor of atherosclerosis and vascular endothelial dysfunction. Injured vascular endothelial cells secrete various inflammatory cytokines including MCP-1 and TNF-α. In addition, it was reported that azelnidipine reduced inflammation [3, 4]. It is also known that various adipocytokines are key molecules for the improvement of urinary albumin excretion [4] and that cohesion of leukocytes on vascular endothelial cell wall is accelerated by VCAM-1 or ICAM-1. Therefore, to explore the mechanism how azelnidipine decreased urinary albumin excretion and carotid atherosclerosis, we evaluated plasma levels of inflammatory cytokines, adipocytokines and soluble adhesion factors. As shown in Fig. 1d, plasma MCP-1 levels were significantly reduced only in azelnidipine group (*p* < 0.05), and TNF-α levels were also reduced only in azelnidipine group (*p* = 0.055). There was no difference between the two groups in the effects on HMW adiponectin and leptin levels (Fig. 1e) and soluble VCAM-1 and ICAM-1 levels (Fig. 1f). These data suggest that azelnidipine affects inflammatory axis rather than adhesion molecules or adipocytokines. We assume that such effects of azelnidipine leads to the reduction of urinary albumin excretion and carotid atherosclerosis.

It is noted here that there is a limitation in this study. First, we compared the effects of azelnidipine and amlodipine, and there was no placebo group in this study. Second, since sample size was small and we did not perform sample size/power calculation in this study, further study with larger sample size would be necessary to strengthen the idea obtained in this study. Finally, since we failed to evaluate smoking habit which is a risk factor to develop atherosclerosis, we cannot exclude the possibility that the difference of the effects between the two drugs was influenced by the difference of smoking habit.

In summary, both azelnidipine (16 mg/day) and amlodipine (5 mg/day) have similar ability to decrease blood pressure in hypertensive patients complicated with type 2 diabetes. However, azelnidipine delayed the progression of urinary albumin excretion and carotid atherosclerosis, which was not observed with amlodipine. We assume that the reduction of inflammation by azelnidipine explains, at least in part, its beneficial effects on urinary albumin excretion and carotid atherosclerosis.

## Abbreviations

CCBs: calcium channel blockers
IMT: intima-media thickness
VCAM-1: vascular cell adhesion molecule-1
ICAM-1: intercellular adhesion molecule-1
